# Trends in hospitalizations for cannabinoid hyperemesis syndrome in Canada, 2016–17 to 2024–25

**DOI:** 10.3389/fpubh.2026.1740300

**Published:** 2026-03-12

**Authors:** Sieara Plebon-Huff, Samantha Goodman, Hanan Abramovici

**Affiliations:** Office of Cannabis Science and Surveillance, Strategic Policy Directorate, Controlled Substances and Cannabis Branch, Health Canada, Ottawa, ON, Canada

**Keywords:** Canada, cannabis, cannabis hyperemesis syndrome, cyclic vomiting, hospitalization

## Abstract

**Objective:**

To evaluate trends in CHS hospitalizations in Canada, including their frequency and individual characteristics.

**Methods:**

This study utilized data from the Canadian Institute for Health Information's Discharge Abstract Database to perform descriptive data analysis and employed JoinPoint Trend Analysis Software to analyze annual percent change (APC) and average annual percent change (AAPC) in hospitalizations for CHS. The main outcome of interest was rate of hospitalization for CHS between fiscal years (FY) 2016–17 and FY 2024–25 (April 2016–March 2025).

**Results:**

There were 7,533 records involving CHS identified in the study period. Overall, mean (SD) patient age was 32.4 (13.1) years, with 3,892 individuals (50.8%) aged 25–44 years, 3,923 (52.1%) males, and 4,205 (55.8%) admitted in Ontario. Overall rates of CHS hospitalizations increased almost 2.5-fold during the nine-year study period, from 1.5 records per 100,000 population in FY 2016–17 to 3.8 per 100,000 in FY 2024–25. Joinpoint analysis identified statistically significant increases across the whole study period (*AAPC* = 13.1, *p*-value = < 0.001), and in both males (*AAPC* = 11.5, *p*-value = < 0.001) and females (*AAPC* = 14.7, *p*-value = < 0.001). For most subpopulations, rates increased significantly from FY 2016–17 to FY 2020–21, with stabilization thereafter. Rates were generally highest among the 20–24 age group for most years. The most rapid increase was noted among individuals aged 45–64 years between FY 2016–17 and FY 2020–21 (*APC* = 27.4, *p*-value = 0.006). Over the whole study period, rates rose continually for two age groups, youth (*APC/AAPC* = 10.8, *p*-value = 0.02) and older adults (*APC/AAPC* = 16.3, *p*-value = 0.01).

**Discussion:**

This repeated cross-sectional study found a significant rise in CHS hospitalizations in Canada between FY 2016–17 and FY 2020–21, followed by a period of stabilization, highlighting key trends in an important cannabis-related health outcome.

## 1 Introduction

Cannabis is one of the most commonly used substances in Canada, with more than one in five (20.7%) Canadians aged 18 years and older reporting past-year use of cannabis in 2024, including 5.6% of Canadians who report daily/almost-daily cannabis use (1). Cannabinoid hyperemesis syndrome (CHS) is a disorder characterized by cyclic, severe nausea and vomiting usually presenting in individuals with chronic, frequent cannabis use, with symptoms frequently relieved by prolonged hot baths or showers (2). First described in 2004 (3), CHS remains underdiagnosed in Canada (4), despite a high prevalence of cannabis consumption and increasing consumption of high potency delta-9-tetrahydrocannabinol (THC)-containing products, like vapes and infused pre-rolled joints (5). Heavy, chronic exposure to high-potency cannabis is thought to contribute to CHS development, and although some theories have been proposed (6) the exact pathophysiological mechanisms remain unknown (7).

Clinically, CHS can be quite impactful and debilitating for patients and families: diagnosis is often delayed, patients typically undergo extensive investigations, and symptom management is largely supportive, with resolution contingent on cessation of cannabis use. In addition, pediatric and adolescent populations are increasingly represented in CHS cases, with studies indicating high recurrence rates and significant healthcare utilization in younger demographic groups (8).

Emerging data from some Canadian provinces indicate a notable increase in emergency visits and care related to CHS (9, 10). However, there are few published data on national-level estimates of CHS hospitalizations. Hospitalization data provide a deeper view of CHS-related harm, its management, and outcomes, and may shed light on the extent to which CHS represents a public health burden in Canada. Given the evolving legal context—including the federal legalization and regulation of cannabis for non-medical purposes in Canada in October 2018—it's also important to identify how these policy shifts may have influenced CHS-related healthcare utilization. The objective of this study was to evaluate trends in CHS hospitalizations in Canada, including their frequency and individual characteristics from fiscal years (FY) 2016–17 to 2024–25 spanning the period of pre-, peri- and post-cannabis legalization and regulation in Canada.

## 2 Methods

### 2.1 Study design, population, and data sources

This study examines cross-sectional population data from the Canadian Institute for Health Information's Discharge Abstract Database (CIHI-DAD), covering all Canadian provinces and territories except Quebec due to differences in collection and reporting systems. The CIHI-DAD records administrative, clinical and demographic information on hospital discharges, including in-hospital deaths, sign-outs and transfers. All hospital discharges from acute care facilities collected between April 1, 2016 and March 31, 2025 (FY 2016–17 to 2024–25) were included.

In accordance with Article 2.2 of the Tri-Council Policy Statement (11), REB review was not required as the analysis used only publicly available, legally protected data.

### 2.2 Outcomes

The primary outcome was annual rates of hospitalizations for CHS per 100,000 population by FY. CIHI added an International Statistical Classification of Diseases and Related Health Problems, Tenth Revision, Canada [ICD-10-CA] code for CHS (K92.81) on April 1, 2022 (12). Prior to this addition, there was no diagnostic code for CHS. To identify CHS hospitalizations, as per previous literature (9, 10, 13), CHS diagnoses were identified as those in which nausea (R11.1), vomiting (R11.2) or nausea and vomiting (R11.3) was the primary diagnosis, and a diagnosis for a mental and behavioral disorder due to the use of cannabinoids (F12-) was recorded during the visit. Only significant diagnoses that influenced the length of hospitalization and/or treatment received were considered (diagnosis types: M, 1, 2, W, X, and Y).

### 2.3 Statistical analysis

Results are broken down by FY, sex, age (average, median and range), age group, length of hospital stay (average, median and range), discharge status, and the province or territory reporting the case. Information on rural vs. urban location and income levels was included for FY 2022–23 onward. All analyses were conducted using SAS Enterprise Guide version 7.1.

Crude rates per 100,000 population were calculated using population estimates from Statistics Canada for July 1 of each year, starting in FY 2016–17 (14). Prevalence estimates for age were adjusted via the direct method using the 2016 Canadian Census population (15).

JoinPoint Trend Analysis Software, version 5.4.0.0 (National Cancer Institute [NCI], Bethesda, MD, USA) (16) was used to examine trends over time from FY 2016–17 to 2024–25. This method looks for points where the trend in rates changes significantly by fitting a straight line to the data and then tests if fit improves with the addition of more “joinpoints”. For this analysis, which included nine data points, the maximum number of joinpoints was set to 1 per NCI guidance (17). Significance tests are performed using a Monte Carlo permutation method (18). Annual percent change (APC) was calculated for each segment, indicating change directionality, with confidence intervals for certainty. Annual average percent change (AAPC) was calculated for the whole study period. Trend analyses were conducted for CHS hospitalizations overall, and by sex and age group. Finally, supplementary analysis using a generalized linear mixed model was conducted to examine the statistical significance of year-over-year changes in rates of CHS hospitalizations (PROC GLIMMIX in SAS EG 7.1). Pairwise comparisons among FY LS-means were performed using Tukey's method.

## 3 Results

### 3.1 Demographics

Overall, this study identified 7,533 hospitalizations attributed to CHS between April 1, 2016 and March 31, 2025. Characteristics of CHS hospitalizations are summarized in Table 1. Mean (SD) patient age was 32.4 (13.1) years, with most records involving individuals aged 25–44 years (50.8%, *n* = 3,829) and males (52.1%, *n* = 3,923). Mean (SD) length of stay was 3.6 (8.4) days, with the majority staying 7 days or less (92.7%, *n* = 6,982), and most hospitalizations were discharged home (85.2%, *n* = 6,416). Over half of the hospitalizations for CHS occurred in Ontario (55.8%, *n* = 4,205). By rurality (FY 2022–23 onward), over a third of CHS hospitalizations occurred in urban regions (35.6%, *n* = 2,682), and 7.4% occurred in rural regions (*n* = 556). By income (FY 2022–23 onward), the largest share of records was attributed to individuals from the lowest income quintile (14.6%, *n* = 1,103), whereas the highest income quintile was associated with the smallest share of CHS hospitalizations (4.4%, *n* = 334).

**Table 1:** Characteristics (counts) of cannabinoid hyperemesis syndrome (CHS) hospitalizations in Canada (excluding Quebec), fiscal year (FY) 2016–17 to 2024–25.

**Variable**	***n* (%)**
Total	7,533 (100)
**Sex**	
Males	3,923 (52.1)
Females	3,609 (47.9)
**Age, years**	
Mean (standard deviation)	32.4 (13.1)
Median	29
Range	5–95
**Age group**	
0–14 years	57 (0.7)
15–19 years	1,015 (13.5)
20–24 years	1,331 (17.7)
25–44 years	3,829 (50.8)
45–64 years	1,127 (15.0)
65+ years	174 (2.3)
**Length of stay (days)**	
Mean (standard deviation)	3.6 (8.4)
Median	2
Range	1–478
**Discharge disposition**	
Died	5 (0.1)
Discharged home	6,416 (85.2)
Transferred	195 (2.6)
Left facility	917 (12.2)
**Submitting province/territory**	
Alberta	1,055 (14.0)
British Columbia	1,198 (15.9)
Manitoba	185 (2.5)
New Brunswick	163 (2.2)
Newfoundland and Labrador	108 (1.4)
Territories (Northwest Territories, Nunavut, Yukon)	82 (1.1)
Nova Scotia	114 (1.5)
Ontario	4,205 (55.8)
Prince Edward Island	89 (1.2)
Saskatchewan	334 (4.4)
**Rurality** ^a^	
Urban	2,682 (35.6)
Rural/remote	556 (7.4)
Unknown/missing	4,285 (56.9)
**Neighborhood income quintile** ^a^	
Lowest quintile	1,103 (14.6)
Medium-low quintile	735 (9.8)
Medium quintile	590 (7.8)
Medium-high quintile	470 (6.2)
Highest quintile	334 (4.4)
Unknown/missing	4,661 (61.9)

^a^Variable available from FY 2022–23 onward.

### 3.2 Trends over time

**Figure 1:**
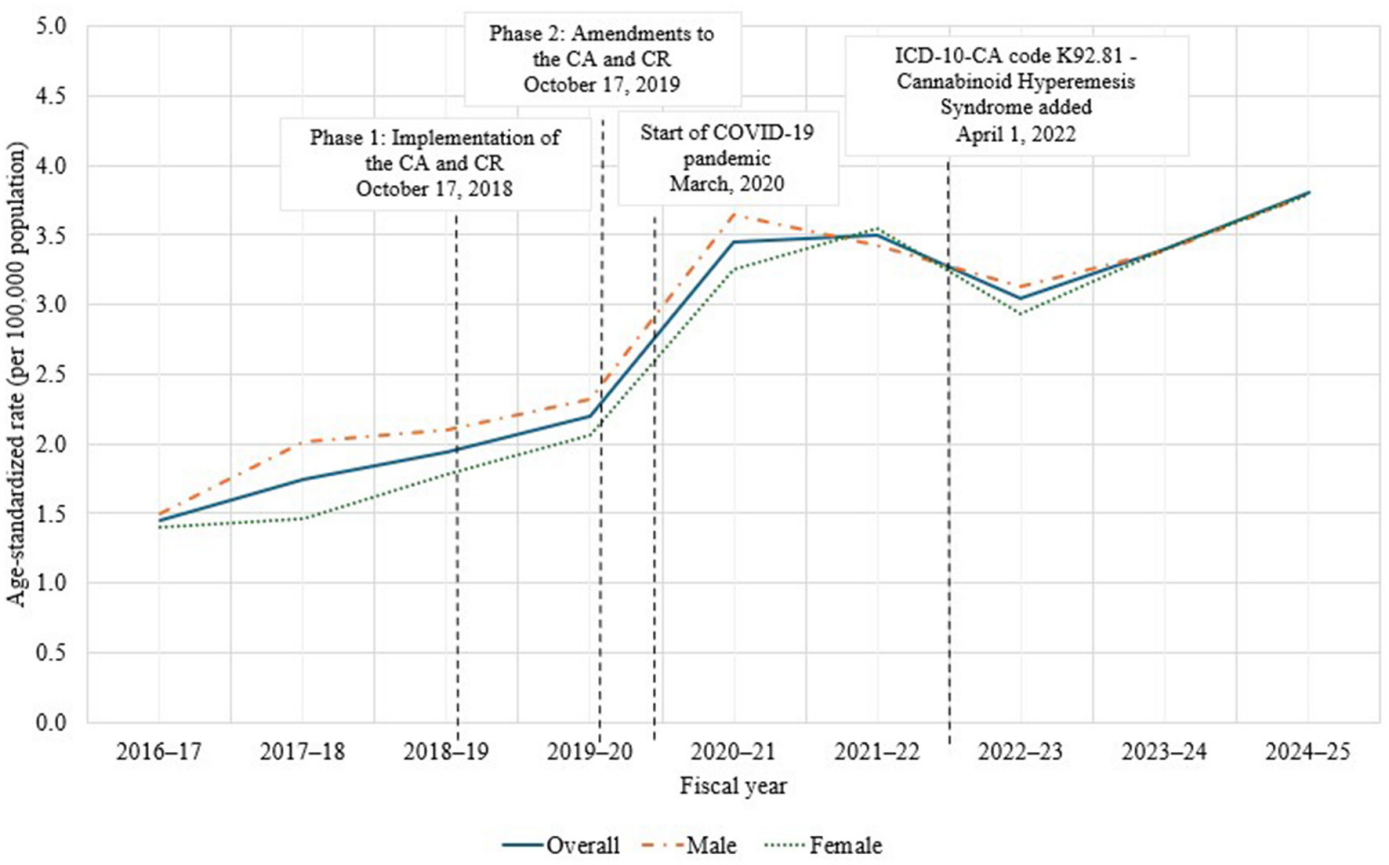
Age-standardized rate (per 100,000 population) of cannabinoid hyperemesis syndrome (CHS) hospitalizations in Canada (excluding Quebec), fiscal year (FY) 2016–17 to 2024–25.

Figure 1 shows the age-standardized rate (per 100,000 population) of CHS hospitalizations across the study period by sex. Overall, the rate of CHS hospitalizations increased over the study period, from 1.5 per 100,000 population in FY 2016–17 to 3.8 per 100,000 population in FY 2024–25, with rates stabilizing at 3.5 per 100,000 population between FY 2020–21 and 2021–22. The age-standardized rate for CHS in males was higher than females from FY 2016–17 to 2020–21, with the opposite pattern observed in FY 2021–22, and rates overlapping for males and females from FY 2023–24 onward. Joinpoint analysis (Table 2) confirmed a statistically significant increase in the rate of CHS hospitalizations overall from FY 2016–17 to 2020–21 (*APC* = 23.5, *p*-value = < 0.001). In contrast, the increase from FY 2020–21 to 2024–25 was not significant, suggesting a stabilization in the overall rate of CHS hospitalizations during the latter portion of the study period. The AAPC across the study period was found to be 13.1% (*p*-value = < 0.001). Supplementary analyses produced a similar pattern of results (Supplement 1). FY had a statistically significant overall effect on rate of CHS hospitalizations (*p*-value = < 0.0001). Pairwise comparisons indicated significant increases year-over-year from FY 2016–17 to 2020–21 (range = 1.5–3.5; *p*-value = < 0.05 for all), with no change from FY 2020–21 to FY 2021–22 (*p*-value = 0.10). Between FY 2021–22 and 2024–25, year-over-year increases and decreases were observed within a fairly tight range (3.0–3.8 per 100,000 population; *p*-value = < 0.001 for all), suggesting a leveling off during this time.

**Table 2:** Joinpoint regression results showing annual percent change (APC) and average annual percent change (AAPC) in cannabinoid hyperemesis syndrome (CHS) hospitalization rates overall, and by sex and age groups, Canada (excluding Quebec), fiscal year (FY) 2016–17 to 2024–25.

**Group**	**Segment**	**Fiscal year**	**Trend direction**	**APC (% per year)**	**95% CI (%)**	***P*-value**	**AAPC (% per year)**	**95% CI (%)**	***P*-value**
Overall	1	2016–2020	Increasing	+23.5^*^	15.6–61.5	< 0.001	—	—	—
	2	2020–2024	Stable	+3.6	−10.9–10.0	0.451	—	—	—
	Overall	2016–2024	Increasing	—	—	—	+13.1^*^	9.0–20.2	< 0.001
**Sex**									
Males	1	2016–2020	Increasing	+21.5^*^	13.4–66.5	0.001	—	—	—
	2	2020–2024	Stable	+2.4	−14.2–9.0	0.562	—	—	—
	Overall	2016–2024	Increasing	—	—	—	+11.5^*^	7.0–19.2	< 0.001
Females	1	2016–2020	Increasing	+25.6^*^	5.2–121.7	0.019	—	—	—
	2	2020–2024	Stable	+4.8	−26.4–24.7	0.427	—	—	—
	Overall	2016–2024	Increasing	—	—	—	+14.7^*^	6.7–30.1	< 0.001
**Age group (years)**									
0–14	Overall	2020^a^-2024	Stable	—	—	—	+3.2	−6.4–13.8	0.511
15–19	Overall	2016–2024	Increasing	—	—	—	+10.8^*^	2.1–22.7	0.018
20–24	1	2016–2020	Increasing	+17.9^*^	12.1–39.7	< 0.001	—	—	—
	2	2020–2024	Stable	+4.0	−6.8–8.8	0.300	—	—	—
	Overall	2016–2024	Increasing	—	—	—	+10.8^*^	7.8–15.2	< 0.001
25–44	1	2016–2020	Increasing	+24.6^*^	16.9–52.5	< 0.001	—	—	—
	2	2020–2024	Stable	+2.5	−8.5–8.5	0.515	—	—	—
	Overall	2016–2024	Increasing	—	—	—	+13.0^*^	9.5–18.4	< 0.001
45–64	1	2016–2020	Increasing	+27.4^*^	9.1–113.4	0.006	—	—	—
	2	2020–2024	Stable	+4.2	−22.4–19.5	0.539	—	—	—
	Overall	2016–2024	Increasing	—	—	—	+15.2^*^	8.0–28.8	< 0.001
65+	Overall	2017^b^-2024	Increasing	—	—	—	+16.3^*^	3.7–35.3	0.013

^*^Indicates that the APC is significantly different from zero at the alpha = 0.05 level.

—Not applicable.

^a^Data prior to 2020 for the 0–14 age group were suppressed due to small cell counts (< 5 cases).

^b^Data prior to 2019 for the 65+ age group were suppressed due to small cell counts (< 5 cases).

Regarding sex, similar trends were observed for males and females. A significant change in the rate of CHS hospitalizations was identified from FY 2016–17 to 2020–21 for both sexes (male: *APC* = 21.5, *p*-value = 0.001; female: *APC* = 25.6, *p*-value = 0.02), with stabilization observed from FY 2020–21 onward (male: *APC* = 2.4, *p*-value = 0.56; female: *APC* = 4.8, *p*-value = 0.43). Despite a higher overall rate of CHS hospitalizations in males, the rate of change from FY 2016–17 to 2020–21 was highest in females, with rates increasing by an average of 25.6% per year during this segment, compared to an increase of 21.5% in males. However, overlapping confidence intervals for males and females during this period (i.e., FY 2016–17 to 2020–21) suggest that this difference is not significant.

**Figure 2:**
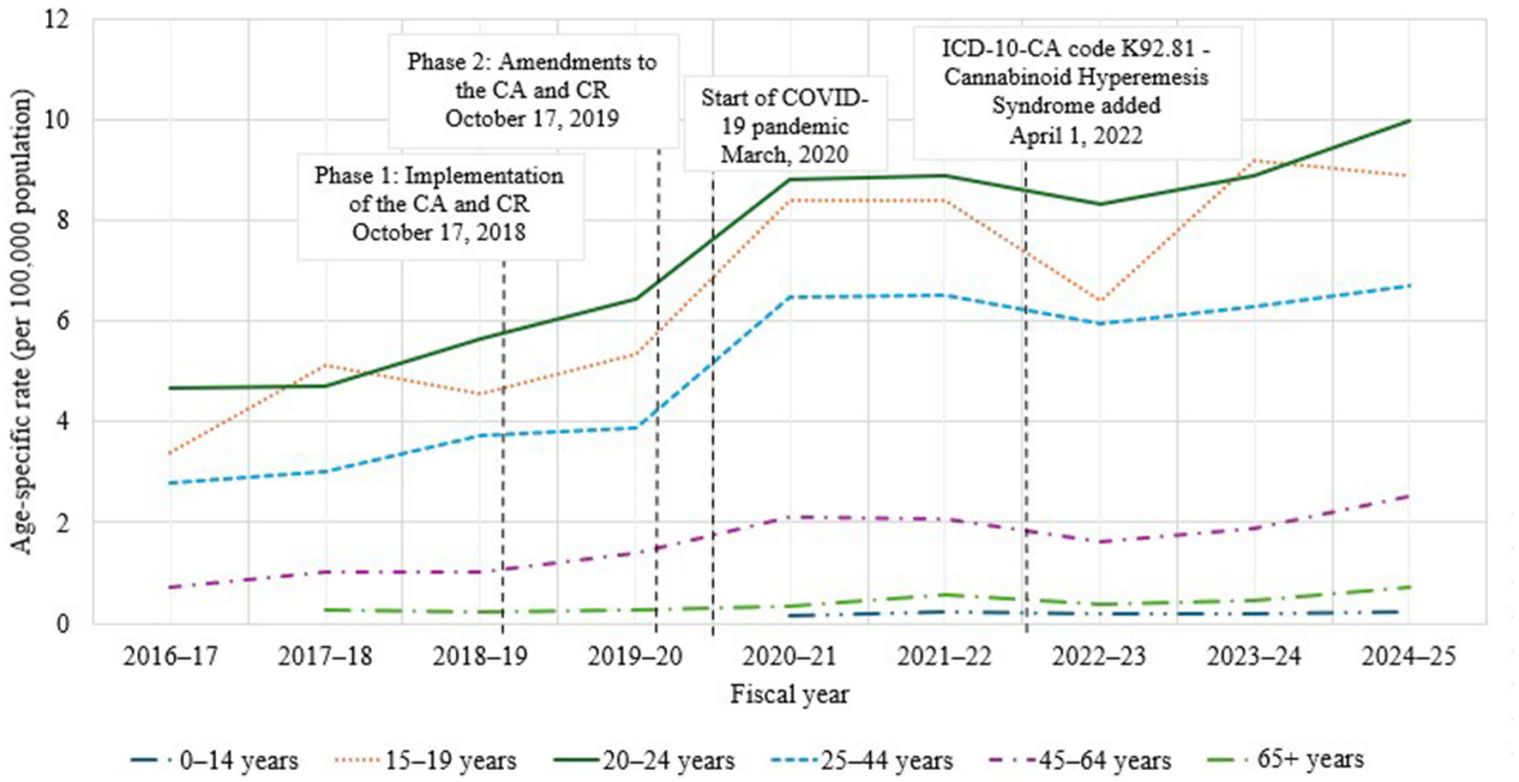
Age-specific rate (per 100,000 population) of cannabinoid hyperemesis syndrome (CHS) hospitalizations in Canada (excluding Quebec), fiscal year (FY) 2016–17 to 2024–25.

Figure 2 shows the age-specific rate (per 100,000 population) of CHS hospitalizations across the study period. The rate of CHS hospitalizations was generally the highest among the 20–24 age group for most years, followed by the 15–19 age group. Rates increased for all age groups across the study period. For the 0–14 age group, rates were calculated for the period of FY 2020–21 to 2024–25 due to small cell sizes prior to FY 2020–21. Joinpoint analysis indicated no statistically significant increase in the rate of CHS hospitalizations for this age group for this period (*APC* = 3.2, *p*-value = 0.51). Joinpoint analysis indicated a statistically significant increase in the rate of CHS hospitalizations from FY 2016–17 to 2024–25 among youth aged 15–19 years (*APC* = 10.8, *p*-value = 0.02).

For the 20–24 age group, a significant and rapid increase was identified from FY 2016–17 to 2020–21 (*APC* = 17.9, *p*-value = < 0.001), followed by stabilization from FY 2020–21 to FY 2024–25 (*APC* = 4.0, *p*-value = 0.30). Similar trends were observed for the 25–44 age group (FY 2016–17 to 2020–21: *APC* = 24.6, *p*-value = < 0.001; FY 2020–21 to 2024–25: *APC* = 2.5, *p*-value = 0.51), and for the 45–64 age group (FY 2016–17 to 2020–21: *APC* = 27.4, *p*-value = 0.006; FY 2020–21 to 2024–25: *APC* = 4.2, *p*-value = 0.54).

Rates for FY 2016–17 for the 65+ age group were excluded due to small cell sizes. However, a statistically significant increase in rates of CHS hospitalizations in this group were identified for the period of FY 2017–18 to 2024–25 (*APC* = 16.3, *p*-value = 0.01).

Notably, the 45–64 age group had the most rapid increase in rates of CHS hospitalizations, increasing by an average of 27.4% per year from FY 2016–17 to 2020–21. Though rates of CHS hospitalizations have been increasing significantly in youth (15–19 years), the rate was slower, at an average of 10.8% per year from FY 2016–17 to 2024–25.

## 4 Discussion

To our knowledge, this is the first publication examining national trends in rates of CHS hospitalizations in Canada. This study provides a comprehensive analysis of hospitalizations due to CHS in Canada over a nine-year period from April 1, 2016 to March 31, 2025 and spanning the period before, during and after legalization and regulation of cannabis for non-medical purposes. These findings indicate that the rate of CHS hospitalizations in Canada has increased 2.5-fold (from 1.5 per 100,000 population in FY 2016–17 to 3.8 per 100,000 population in FY 2024–25). Rates of CHS hospitalizations were highest in youth and young adults, individuals earning lower incomes, and in males overall, which is consistent with patterns of daily/almost-daily cannabis use among Canadians (2, 5). These data also demonstrate a rapid increase in the rate of CHS hospitalizations for most subgroups prior to legalization and regulation of cannabis for non-medical purposes. In some subgroups, rates appeared to stabilize from FY 2020–21 onward, coinciding with continued expansion in the legal cannabis market and increased diversification in the types of cannabis products being consumed.

The demographic profile of individuals hospitalized for CHS was consistent with prior literature, with the majority being young adults (mean 32.4 years), and a general predominance among males (9, 19, 20). The predominance in urban settings and lower-income populations has previously been noted (9), and may suggest potential sociodemographic factors influencing CHS risk and healthcare access. The longer length of stay variability, indicated by the large standard deviation (8 days), may reflect heterogeneous clinical presentations and possibly varying management strategies for CHS (21). However, only 7% of CHS hospitalizations were longer than 7 days. The disproportionate representation of individuals from lower-income quintiles underscores potential health inequities related to cannabis-related harms. This socioeconomic gradient may be linked to differential cannabis use patterns (22), access to healthcare, or other social determinants of health, warranting further investigation.

Sex-specific trends revealed interesting shifts over time. Initially, males exhibited higher hospitalization rates; however, from FY 2021–22 onward, rates in females caught up and eventually overlapped with males by FY 2023–24. The APC from FY 2016–17 to 2020–21 was significantly greater in females compared to males, suggesting a rapidly growing burden of CHS among females. This may reflect changing patterns of cannabis use between sexes, evolving awareness, or differences in health-seeking behavior (23–25).

Age-specific analyses highlighted the highest rates of CHS hospitalizaitons among young adults aged 20–24 years, followed by youth aged 15–19 years, consistent with prior findings linking younger age groups to higher cannabis-related harms more generally (26). However, significant increases were observed in older adults (65+ years), albeit with different magnitudes. The rapid increase in hospitalizations among older adults is a novel and noteworthy finding, indicating that CHS may be increasingly affecting older adults. This demographic shift may be attributable to increased social acceptability and destigmatization of cannabis use, increased product potency, or more chronic usage patterns in older populations (27–31). Further research is needed to elucidate factors contributing to these observations.

Although there were more rapid increases in rates of CHS hospitalizations among other age groups, the observed increase among youth is a concern. Beyond acute symptoms of CHS, chronic cannabis use, which often precipitates CHS, is detrimental to adolescent brain development. The average age of first-time cannabis use is 14 years old in Canada (32). This early initiation raises significant concerns about the vulnerability of the developing brain to cannabis-related harms. Cannabis use during adolescence has been linked to alterations in brain structure and function, including impaired cognitive abilities, reduced IQ and increased risk for neuropsychiatric outcomes, particularly with use of high-potency products (33–40). The increase in CHS hospitalizations among youth suggests an even more concerning pattern of frequent, chronic cannabis use, which not only exacerbates gastrointestinal distress but also signals potentially harmful long-term neurodevelopmental consequences.

While rates of CHS hospitalizations appeared to rise around the segments corresponding to phase 1 (October 17, 2018; availability of dried cannabis, fresh cannabis and ingestible oils) and phase 2 (October 17, 2019; availability of extracts for inhalation, edibles and topicals) of legalization and regulation of cannabis (see Figures 1, 2), a joinpoint was not detected at these specific timepoints. However, these timepoints do fall within a segment characterized by a statistically significant increasing trend. This suggests that the increases observed at phase 1 and phase 2 of legalization are part of a broader upward trend rather than a new or sudden change in trend at that specific time. Indeed, supplementary analysis demonstrated statistically significant increases in rates of CHS hospitalizations for all FY intervals analyzed, except between FY 2020–21 and FY 2021–22. Moreover, a significant decline in hospitalization rates was observed between FY 2021–22 and FY 2022–23.

Stabilization of hospitalization rates from FY 2020–21 to 2024–25 across most subgroups may indicate that, despite a rise in case counts, the proportion of individuals requiring inpatient care has remained steady. This trend could reflect improved outpatient management strategies, shifts in the severity or demographics of cases, or changes in healthcare utilization patterns influenced by the COVID-19 pandemic (41). For instance, Canadian data suggest that during the COVID-19 pandemic, some individuals were reluctant to seek hospital care (42, 43). Similarly, in FY 2022–23, ICD-10-CA code K92.81 was introduced. New diagnostic codes often raise awareness and update clinical guidelines, potentially impacting reported rates through better identification. However, a distinct code can improve accuracy by reducing risk of misclassification. It is not immediately clear how the implementation of K92.81, and growing awareness of CHS, have impacted the rate of CHS hospitalizations in Canada. Continued surveillance is necessary to monitor whether these trends persist as cannabis regulation, policies, and public health initiatives evolve.

This study has important clinical and public health implications. The rising burden of CHS hospitalizations highlights the need for increased awareness among healthcare providers to recognize and manage CHS effectively. Public health messaging should continue to address the risks of chronic cannabis use, especially in vulnerable populations identified in this study. Future research could explore causal pathways, preventive strategies, and the impact of cannabis product characteristics, including potency, on CHS risk.

### 4.1 Limitations

This study has several limitations due to the use of health administrative data. CHS case identification relies on diagnostic codes, which may be misclassified or underreported, especially given the potential lack of familiarity with CHS and variability in coding practices as well as changes in coding over time. In fact, the presence of CHS diagnoses in children (< 5 records) further suggests potential misclassification or miscoding given that CHS occurs in individuals with chronic, frequent cannabis use, which is not typically observed in children under the age of 12. Secondary analysis indicated that the use of combination codes as a proxy for CHS (i.e., F12. - plus R11.1, R11.2, and/or R11.3) continued at low levels after FY 2021–22. This suggests that some misclassification of codes persisted despite the widespread adoption of K92.81. Excluding this proxy coding after FY 2021–22 may have led to an underestimation of CHS hospitalizations. More training and awareness on proper and consistent coding practices for CHS could assist in preventing future miscoding and misclassification.

In addition, the data used in this analysis lack clinical details such as cannabis use patterns, source of products, product types, or symptom severity, which limit analysis of causality. Cases treated outside of hospitalization may be missed, underestimating the true burden. Trends may be affected by changes in healthcare-seeking behavior, clinician awareness, or policy changes over time (e.g., initial announcement of intention to legalize cannabis for non-medical purposes, launch of the Task Force on Cannabis Legalization and Regulation, implementation of phase 1 and phase 2 of legalization). This study does not account for other confounding factors like concurrent substance use or comorbidities. Similarly, the impact of the COVID-19 pandemic is unknown and could have confounded the stabilization in rates observed after FY 2020–21.

Despite these limitations, health administrative data provide valuable insights into temporal trends and characteristics related to CHS hospitalizations in Canada, highlighting the need for further research incorporating detailed clinical and behavioral data.

## 5 Conclusions

These findings reveal a significant increase in the rate of CHS-related hospitalizations early in the study period before legalization and regulation of cannabis for non-medical purposes, with notable demographic shifts and stabilization at higher rates in more recent years after legalization and regulation. These trends illustrate how the public health consequences of cannabis use continue to evolve and underscore the importance of ongoing surveillance to effectively monitor, understand, and respond to the burden of CHS and other cannabis-related harms.

## Data Availability

The data analyzed in this study are subject to the following licenses/restrictions: The data that support the findings of this study are available from the Canadian Institute for Health Information (CIHI). Restrictions apply to the availability of these data, which were used under a data access agreement for the current study. Data are not publicly available but may be accessed by qualified researchers, decision makers or health managers through a formal request process to CIHI. Requests to access these datasets should be directed to https://www.cihi.ca/en/access-data-and-reports/data-holdings/make-a-data-request.
