# Supplementary material for: Trends in hospitalizations for cannabinoid hyperemesis syndrome in Canada, 2016–17 to 2024–25
Source: Front Public Health. 2026 Mar 12;14:1740300. doi: 10.3389/fpubh.2026.1740300 (PMC13017946; doi:10.3389/fpubh.2026.1740300)
Supplement: Supplementary file 1 [file Table_1.docx]

Supplementary Material

# Supplementary Analysis of Year-Over-Year Changes in Rates of Cannabis Hyperemesis Syndrome Hospitalization

## Purpose

To determine the change in rates of CHS hospitalization year-over-year, and whether these changes are statistically significant. This secondary analysis was conducted to supplement the original analysis in the manuscript, which uses a more robust methodology to identify key changes in the data over the whole study period.

## **Methods**

A generalized linear mixed model (GLMM) was used to evaluate the effect of fiscal year (FY) on the rate of CHS hospitalization and determine the statistical significance of the change in rate year-over-year. The analysis was performed using the PROC GLIMMIX procedure in SAS EG 7.1.

- Model specification: The outcome variable was the count of cases out of the total population for each FY, modeled with a binomial distribution and logit link function.
- Fixed effects: FY was included as a categorical fixed effect.
- Estimation: Least squares means (LS-means) of the FY effect were computed to estimate the mean proportion of cases for each FY.
- Multiple comparisons adjustment: Pairwise comparisons among FY LS-means were performed using Tukey’s method to control for type I error due to multiple testing.
- Outputs: The model provided adjusted p-values for differences between FYs, along with confidence intervals for the LS-means differences.

## Results

Fiscal year has a statistically significant overall effect on the rate of CHS hospitalization (p = <.0001). Pairwise comparisons indicate significant changes in the rate of CHS hospitalization year-over year, except from FY 2020-21 to FY 2021-22.

**Supplementary Table 1.** Year-Over-Year Changes in Rates of Cannabis Hyperemesis Syndrome Hospitalization

| Fiscal year | Overall rate of CHS hospitalization  (per 100,000 population) | Relative change in rate (%) | Statistically significant from previous year (Yes/No, adjusted p-value) |
| --- | --- | --- | --- |
| 2024–25 | 3.8 | +11.6 | Yes (<.0001) |
| 2023–24 | 3.4 | +12.0 | Yes (<.0001) |
| 2022–23 | 3.0 | -13.1 | Yes (.0008) |
| 2021–22 | 3.5 | +1.3 | No (.9999) |
| 2020–21 | 3.5 | +57.0 | Yes (<.0001) |
| 2019–20 | 2.2 | +13.3 | Yes (.0039) |
| 2018–19 | 1.9 | +11.3 | Yes (0.0273) |
| 2017–18 | 1.7 | +19.9 | Yes (<.0001) |
| 2016–17 | 1.5 | - | - |
